# Supplementary material for: Multi-channel ultrasonic Bessel vortex beams by spatial multiplexing metalens
Source: Commun Eng. 2026 Feb 6;5:50. doi: 10.1038/s44172-026-00599-3 (PMC12988197; doi:10.1038/s44172-026-00599-3)
Supplement: Supplementary file 2 — Supplementary Information for Multi-channel ultrasonic Bessel vortex beams by spatial multiplexing metalens [file 44172_2026_599_MOESM2_ESM.pdf]

Supplementary Information for

Multi-channel ultrasonic Bessel vortex beams by spatial multiplexing metalens

Yinjie Su[a], Di Wang[a], Zhongming Gu[a,*], Chen Liu[a] and Jie Zhu[a,*]

[a] *Institute of Acoustics, School of Physics Science and Engineering, Tongji University, Shanghai 200092, People's Republic of China*

* *Corresponding author: zhmgu@tongji.edu.cn; jiezhu@tongji.edu.cn*

Supplementary Note 1. The generation of Bessel beams

The generation of Bessel beams requires precise phase modulation through acoustic lenses to control the direction of transmitted wave. Specifically, the lens configuration induces wavefront tilting along the axial direction, enabling the transmitted wave to interfere with itself and form the characteristic Bessel beam pattern, as shown in Supplementary Fig. 1. The phase profile of the lens can be expressed as

$$\varphi = k_0 \sin\alpha \sqrt{x^2 + y^2} \tag{1}$$

where $k_0 = 2\pi f / c_0$ represents the wavenumber in water.

It should be noted that theoretically producing ideal Bessel beams necessitates acoustic sources and lenses with infinite aperture sizes. In practical implementations, the aforementioned method inevitably generates truncated Bessel beams (truncated Bessel beams), whose effective length L exhibits a functional dependence on the wavefront tilt angle α. Excessive tilt angles (α > 12°) result in diminished interference regions, while insufficient tilt angles (α < 12°) lead to suboptimal beam intensity despite potential extension of the effective interaction length. Through systematic parameter optimization and experimental verification, we determined that selecting α = 12° achieves an optimal balance between maintaining sufficient beam intensity and maximizing the effective propagation length of truncated Bessel beams.

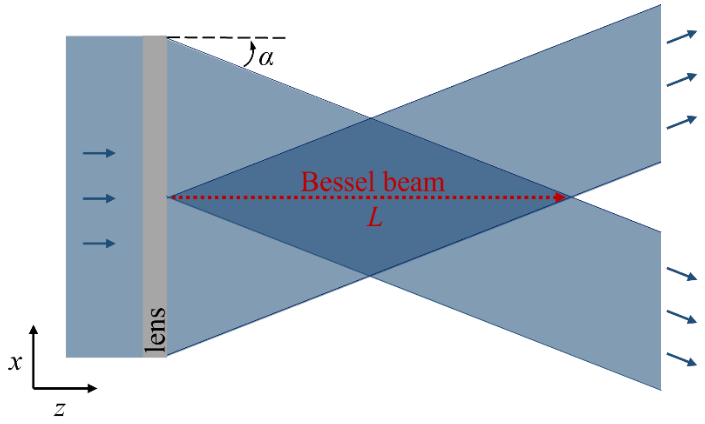

Supplementary Figure 1. Schematic diagram of truncated Bessel beams in the interference region by tilting the axial waves axial direction.

Supplementary Note 2. Comparison between spatial multiplexing scheme and traditional phase superposition scheme

We selected a simplified case to demonstrate the operational differences between spatial multiplexing and conventional phase superposition schemes in phase-only holograms. Following the principles of these two distinct approaches, dual wave beams were generated at ±θ relative to the *x*-axis, with their respective actuation phases corresponding to

$$\varphi_1 = \arg\left(exp\left(-ik_0 ysin(\theta)\right)\right) \tag{2}$$

$$\varphi_2 = \arg\left(exp\left(ik_0 ysin(\theta)\right)\right) \tag{3}$$

According to the phase superposition scheme, the phase distribution of the sound source can be expressed as

$$\varphi_s = \arg\left(exp\left(-ik_0 ysin(\theta)\right) + exp\left(ik_0 ysin(\theta)\right)\right) \quad (4)$$

Three representative tilt angles (θ = 30°, 15°, 10°) were systematically analyzed to compare the theoretical performance between the two modulation schemes. Figures S2a-c present the computational results of spatial multiplexing scheme under these angular configurations, while Figures S2d-f display the corresponding outcomes from phase superposition scheme. Notably, the phase superposition approach exhibits significant higher-order diffraction components in the modulated acoustic field, whereas the spatial multiplexing method does not generate such artifacts.

To elucidate this phenomenon, the source configurations for both schemes are comparatively presented in Fig.S3. In the phase superposition scheme (Figures S3a, b), the achieved amplitude profile (solid line in S3a) deviates from the ideal superimposed field distribution (dashed line) due to the intrinsic inability of phase-only holograms to reconstruct amplitude information. The corresponding phase distribution is shown in Supplementary Fig. 3b. Conversely, the spatial multiplexing scheme does not require amplitude control (Supplementary Fig. 3c), relying solely on spatially interleaved phase distributions (φ_1 and φ_2) as demonstrated in Supplementary Fig. 3d, where solid dots and open circles represent discretely arranged phase elements.

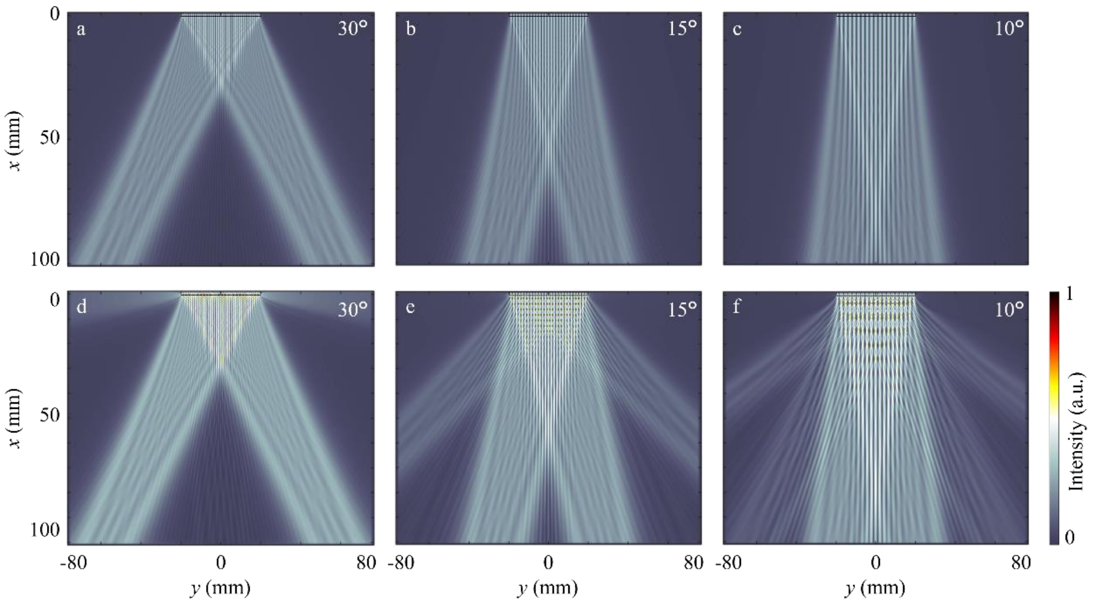

Supplementary Figure 2. Schematic diagram of the sound field generated by the source encoded by spatial multiplexing scheme and traditional phase superposition scheme. a-c The sound beam generated by spatial multiplexing scheme with an angle of 30°, 15°, and 10° with the *x*-axis. **d-f** The sound beam generated by phase superposition scheme with an angle of 30°, 15°, and 10° with the *x*-axis.

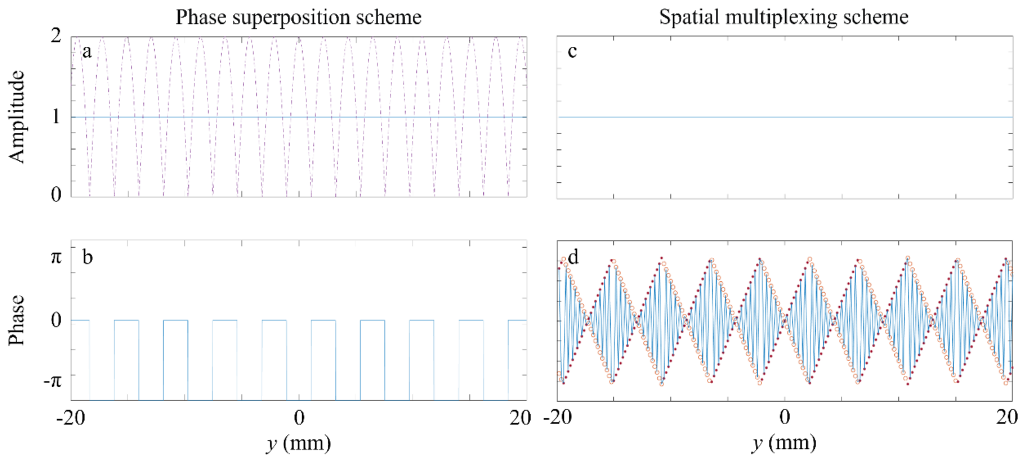

Supplementary Figure 3. Schematic diagram of source setting of spatial multiplexing scheme and phase superposition scheme. a The amplitude distribution (solid line) provided by POH and the amplitude distribution required for the restoration of the sound field (dashed line). **b** The phase distribution generated by phase superposition scheme. **c** The amplitude distribution generated by Spatial multiplexing scheme. **d** The phase interval distribution generated by spatial multiplexing scheme. Points and circles represent the phase distribution of beams propagating in two directions respectively.

Supplementary Note 3. Comparison of simulated acoustic fields between spatial multiplexing and phase superposition schemes.

To provide a quantitative comparison between the spatial multiplexing scheme and the conventional phase superposition method, we conducted additional 3D numerical simulations. Both schemes were configured to generate four vortex beams deflected at 6° relative to the z-axis. This specific tilt angle was chosen to make the differences in acoustic field distribution between the two schemes more pronounced.

The acoustic intensity distributions on the *xoy* plane for both schemes are presented in Supplementary Fig. 4. The spatial multiplexing scheme produces cleaner and more distinct beam profiles with reduced spurious sidelobes compared to the phase superposition scheme.

To further quantify the performance, we calculated the energy concentration ratio for each beam, defined as the percentage of acoustic energy contained within the main lobe relative to the total energy in a surrounding region (within a 10-wavelength radius from each main lobe's center). The main lobe energy ratios for the four channels generated by the spatial multiplexing scheme are 60.73%, 62.07%, 70.56%, and 48.27%, respectively. In contrast, the ratios for the phase superposition scheme are 57.01%, 47.62%, 49.38%, and 48.02%. The higher values achieved by the spatial multiplexing scheme demonstrate its superior capability in concentrating acoustic energy into the intended main lobes, thereby improving energy utilization efficiency and enhancing directional decoupling for multi-channel operation.

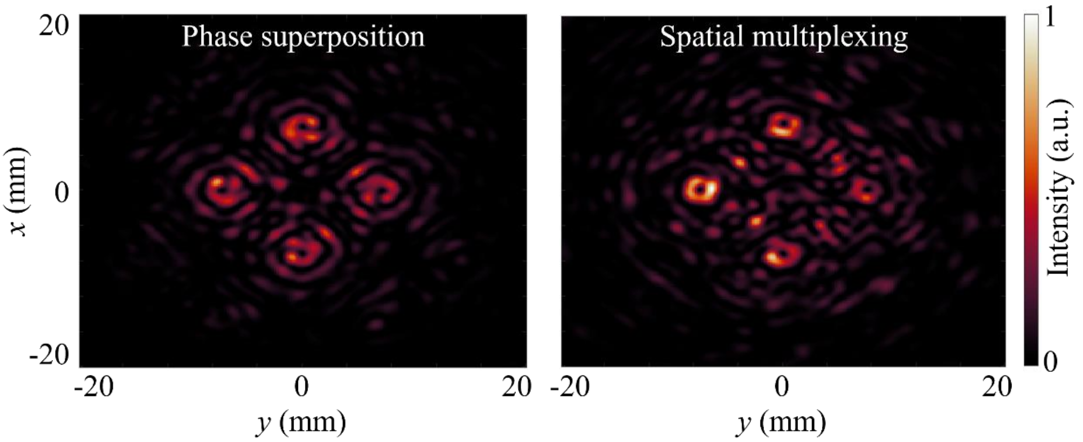

Supplementary Figure 4. Comparison of simulated acoustic fields between spatial multiplexing and phase superposition schemes.

Supplementary Note 4. Far-field intensity distribution of acoustic vortices with different topological charges

To quantitatively evaluate the intensity and attenuation of acoustic vortices of different orders, we conducted additional numerical simulations comparing the performance of vortices with topological charges m = ±1, 3, 5. We calculated the variation of acoustic intensity along the central axis of each vortex beam over a propagation distance from z = 71 mm to z = 96 mm (exceeding 30 wavelengths at 2 MHz).

The results are shown in Supplementary Fig. 5. Higher-order vortices (|m| = 3, 5) have broader annular energy distribution profiles, with their acoustic energy distributed over a larger area, resulting in lower on-axis intensity compared to the fundamental |m| = 1 vortex. The intensity ratios of |m| = 1, 3, and 5 acoustic vortices at the same position are approximately 1:0.72:0.51, indicating that the vortex beam intensity is related to its topological order. Additionally, the intensity of vortices of all orders maintains 60% of its initial value after 25 mm of propagation, demonstrating good propagation stability of the acoustic beams.

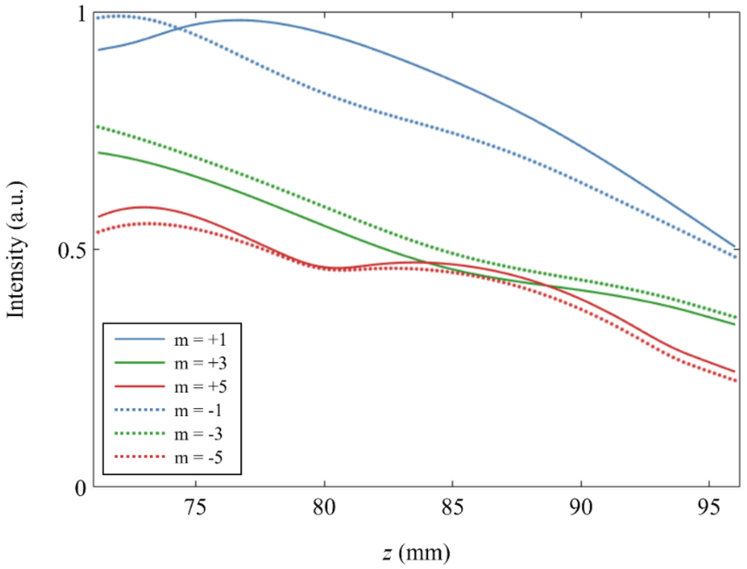

Supplementary Figure 5. Far-field intensity distribution of acoustic vortices with different topological charges. Acoustic intensity distribution curves along the propagation axis for vortices of orders m=+1, +3, +5. The intensity attenuation trends remain consistent for vortices of all orders over the propagation distance from 71 mm to 96 mm.

Supplementary Note 5. Simulated transverse profiles of multiplexed vortices

To provide a direct numerical counterpart to the experimental measurements, and to further validate our design, we performed additional simulations of the acoustic field in the transverse (*xoy*) plane at the same far-field distance (z = 62 mm).

Supplementary Figure 6 displays the simulated acoustic pressure and phase distributions for the two customized metalens designs. Supplementary Figure 6a and b correspond to the two-channel sample (where only Channels 2 and 4 are active, both encoded with m = +1 vortices). The simulated donut-shaped pressure profile and the characteristic 2π-phase spiral in b confirm the successful generation of a high-quality +1-order vortex. Supplementary Figure 6c and d correspond to the high-order vortex sample (with Channels 2 and 4 encoded with m = +3 and m = -1, respectively, as in the main text). The simulated results clearly show the enlarged donut profile of the higher-order (m = +3) vortex and the opposite helicity of the phase for the negative-order (m = -1) vortex. These simulation results (Supplementary Fig. 6) closely match the corresponding experimental measurements (Fig. 5b, d in the main text), confirming the successful generation of the predefined vortices.

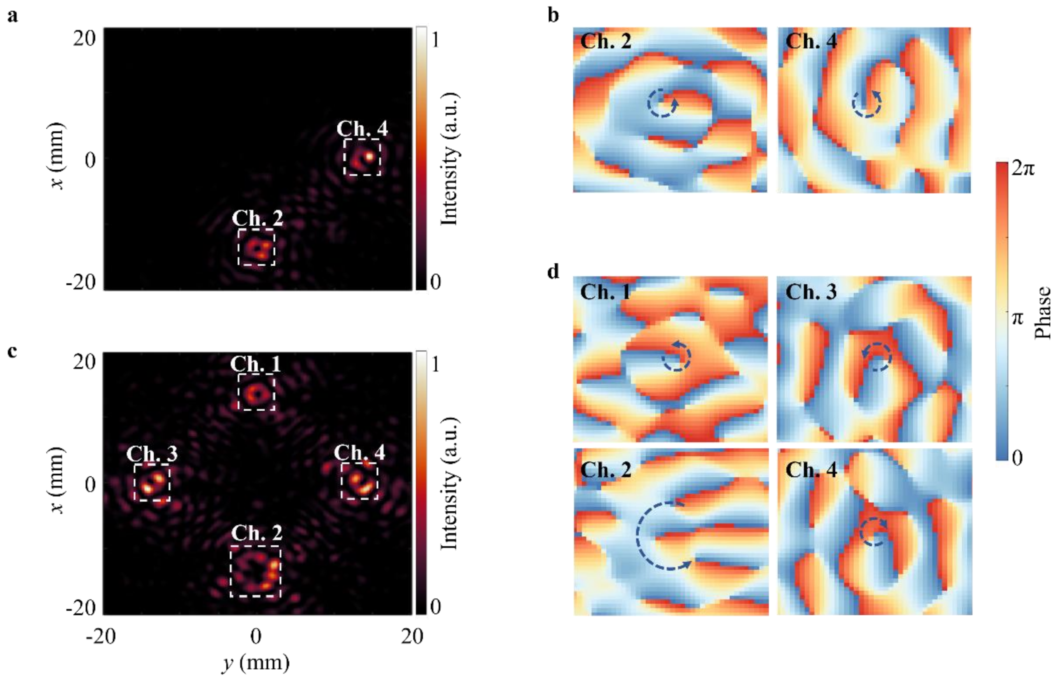

Supplementary Figure 6. Simulated acoustic field distributions in the transverse plane for customized metalenses. a Acoustic pressure and **b** phase distributions generated by the two-channel metalens (Channels 2 & 4, both m = +1) at z = 62 mm. **c** Acoustic pressure and **d** phase distributions generated by the high-order metalens (Channel 2: m = +3, Channel 4: m = -1) at z = 62 mm.

Supplementary Note 6. The relationship between number of channels and sound intensity

To prove the relationship between channel number and acoustic intensity, we performed simulations encoding Bessel vortex beams onto an increasing number of channels, while configuring residual channels with spherical wavefronts to induce rapid energy dissipation. The corresponding phase distributions are given by:

$$\varphi_v = m\theta + k_0 \sin\alpha \sqrt{x^2 + y^2} \quad (5)$$

$$\varphi_s = k_0 \sqrt{x^2 + y^2 + z_0^2} \quad (6)$$

Here, m = 1 and α = 12° are consistent with the main text parameters, while z_0 = 30 mm enables accelerated diffraction for this specific channel. As shown in Supplementary Fig. 7, vortex energy increases progressively with the number of vortex-encoded channels. The intensity ratios scale as 1 : 4.4 : 9.5 : 15.6 across 1-4 channels, exhibiting near-quadratic dependence on channel count.

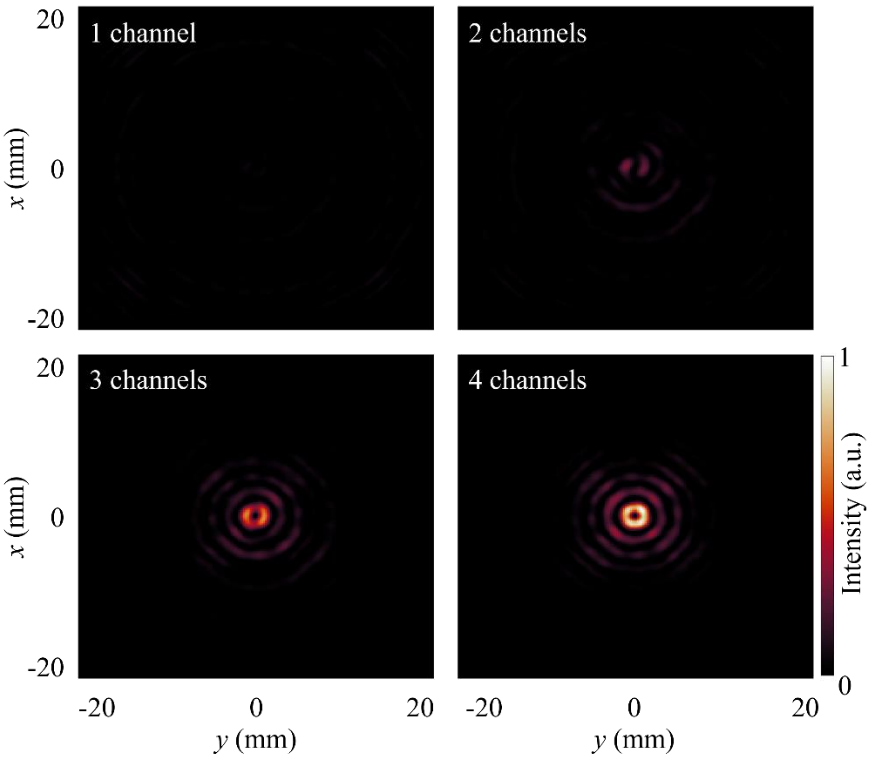

Supplementary Figure 7. Sound intensity increases as vortex-encoded channel number increases.

Supplementary Note 7. Quantitative Analysis of Vortex Beam Modal Purity

In response to the reviewer's suggestion and to quantitatively evaluate the quality of the multi-channel Bessel vortex beams generated by our spatially multiplexed metalens, we calculated their modal purity. This method objectively reflects the mode generation efficiency and inter-channel crosstalk by measuring the degree of match between the generated acoustic field and the target ideal vortex mode.

According to Eq. (1) in the main text, setting α=12°, β=γ=0, we generated ideal Bessel acoustic vortex fields $\Psi_m(x,y)$ of orders from m = -3 to m = +3 as a set of orthogonal bases. The modal purity is calculated based on the modulus of the normalized correlation coefficient between the complex acoustic pressure distribution of the generated field, $P(x,y)$, and the target mode, $\Psi_m(x,y)$. The specific calculation formula is as follows:

$$\text{Purity}(m) = \left| \frac{\iint P(x,y) \cdot \Psi_m^*(x,y)\ dx\ dy}{\sqrt{\iint |P(x,y)|^2\ dx\ dy} \cdot \sqrt{\iint |\Psi_m(x,y)|^2\ dx\ dy}} \right|$$

where Ψ_m^* represents the complex conjugate of the ideal mode. This coefficient essentially calculates the cosine similarity between the two fields, with a value range of [0, 1]. A purity value

closer to 1 indicates a higher degree of match between the generated field $P(x, y)$ and the target mode Ψ_m with topological charge m, meaning higher modal purity and lower crosstalk with other modes.

We analyzed the simulation results for the three metalenses discussed in the main text (uniform +1-order, dual-channel, and high-order) and calculated the modal purity of the vortex beam for each channel. The calculation results are presented as bar charts in Supplementary Fig. 8. The modal purities for the four channels (all preset to m = +1) are 82.18%, 86.37%, 85.14%, and 81.61%, respectively. All channels exhibit high and consistent purity, confirming that the metalens can generate multiple high-quality, low-crosstalk +1-order vortices in parallel. The modal purities for the two active channels (both preset to m = +1) are 80.36% and 76.32%, respectively. The high purity levels demonstrate the effectiveness of the scheme under different channel configurations. The modal purities for the four channels (with preset topological charges of m = +1, +3, +1, −1, respectively) are 85.33%, 82.65%, 76.99%, and 76.41%. The high purity achieved for the higher-order vortices (m = +3 and m = −1) demonstrates the capability of the metalens to generate complex vortex modes.

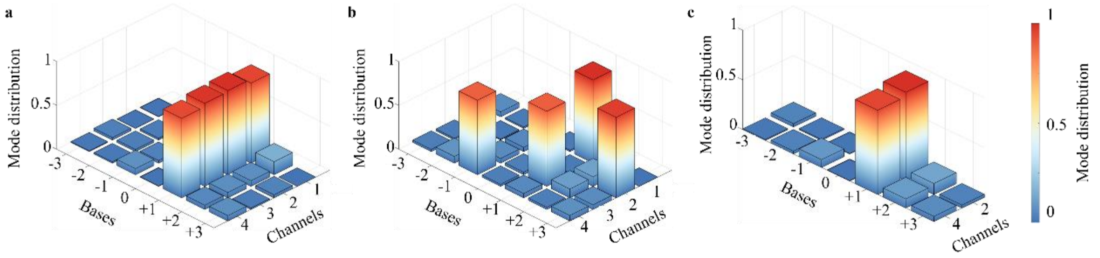

Supplementary Figure 8. Quantitative analysis of the modal purity of vortex beams generated by the metalens under different configurations. a Sample generating m = +1 vortices in all four channels. **b** Sample generating m = +1 vortices in two channels. **c** Sample generating higher-order vortices (Channel 2: m = +3, Channel 4: m = −1).
